# Towards the achievement of universal health coverage in the Democratic Republic of Congo: does the Country walk its talk?

**DOI:** 10.1186/s12913-022-08228-3

**Published:** 2022-07-04

**Authors:** Alexis Biringanine Nyamugira, Adrian Richter, Germaine Furaha, Steffen Flessa

**Affiliations:** 1 Université Evangélique en Afrique, Bukavu, Democratic Republic of the Congo; 2 University of Greifswald, Greifswald, Germany; 3 University Medicine Greifswald, Institute for Community Medicine, Greifswald, Germany

**Keywords:** Universal Health coverage, Financial risk protection, Health outcomes, Health insurance, Out-of-pocket, Democratic Republic of the Congo (DRC)

## Abstract

In 2009, the Democratic Republic of Congo (DRC) started its journey towards achieving Universal Health Coverage (UHC). This study examines the evolution of financial risk protection and health outcomes indicators in the context of the commitment of DRC to UHC. To measure the effects of such a commitment on financial risk protection and health outcomes indicators, we analyse whether changes have occurred over the last two decades and, if applicable, when these changes happened. Using five variables as indicators for the measurement of the financial risk protection component, there as well retained three indicators to measure health outcomes. To identify time-related effects, we applied the parametric approach of breakpoint regression to detect whether the UHC journey has brought change and when exactly the change has occurred.

Although there is a slight improvement in the financial risk protection indicators, we found that the adopted strategies have fostered access to healthcare for the wealthiest quantile of the population while neglecting the majority of the poorest. The government did not thrive persistently over the past decade to meet its commitment to allocate adequate funds to health expenditures. In addition, the support from donors appears to be unstable, unpredictable and unsustainable. We found a slight improvement in health outcomes attributable to direct investment in building health centres by the private sector and international organizations. Overall, our findings reveal that the prevention of catastrophic health expenditure is still not sufficiently prioritized by the country, and mostly for the majority of the poorest. Therefore, our work suggests that DRC’s UHC journey has slightly contributed to improve the financial risk protection and health outcomes indicators but much effort should be undertaken.

## Background

According to the World Health Organization (WHO), health spending remains unequal across countries. The United States alone accounts for 42% of global health spending. More than 60% of global health spending is accounted for France, Germany, Japan, the United Kingdom and the USA [1]. In 2015, the WHO estimated that, globally, around 400 million people lack access to at least one essential health service and that approximately 100 million are impoverished every year because of healthcare costs [2]. Universal Health Coverage (UHC) means that everyone in the population has access to preventive, curative and rehabilitative healthcare when they need it and at an affordable price [3, 4]. UHC aims to ensure that healthcare benefits are distributed based on the need for care and not on the ability to pay [5, 6].

The achievement of UHC requires a commitment to three fundamental principles: (i) mobilizing adequate resources to ensure coverage, (ii) providing quality care through strengthening the health service delivery system and (iii) ensuring that health services are accessible to all impoverished and vulnerable individuals [7]. The protection of people from catastrophic health expenditures is widely accepted as a desirable objective of health policy. Therefore, catastrophic payment from individuals’ available income can drop many households into poverty [8].

Previous findings support that the poor have a higher incidence and higher out of pocket payments so that they are more likely to incur catastrophic health expenditures than the well-off [6, 9–12]. To reduce the incidence of catastrophic health payments, the World Health Organisation recommends that total out-of-pocket expenses should not exceed 15–20% of national health expenditures [8]. Lower-income countries lag behind on the road to UHC, especially many African countries. Although they have experienced good economic growth in the past two decades, improvements in health outcomes have been slow and uneven in many African countries [13]. In 2018, the average government spending on healthcare in lower-income countries was only US$ 9 per capita, representing 1.2% of Gross Domestic Product with a marginal contribution of social health insurance which was only greater than US$ 5 per capita in some countries [1].

In Africa, health-financing strategies vary broadly by geographic region and social context. According to [14] the notable differences between countries regarding their health financing strategies show how health systems are influenced by social, cultural, economic and political factors resulting from the country’s context-specific. Although Universal Health Coverage (UHC) has become a political priority for many African countries, it has been challenging to achieve.

In the Democratic Republic of Congo, per capita, health expenditure remains low and largely below what other low-income countries have invested [15]. According to [16], at USD 13 per capita, DRC spends less than one-tenth the average of the rest of sub-Saharan Africa on health. In 2019, the country has allocated only 3.5% of the GDP and 8.5% of its budget to health financing. At a rate of 846 deaths per 100.000 births over 2007–2014, DRC’s rate of maternal mortality was higher than the average of the sub Saharan Africa of 510 [16]. In their research, [17] also emphasised the need for well-trained human resources for health who do not meet international standards in DRC’s rural areas. In the same rural areas, health facilities encounter large shock-outs of essential drugs and the population travel long distance to attend health facilities. Therefore, the financing gap for health is high as the country aims to achieve the UHC strategic objectives [17].

Out-of-pocket payments account for more than 90% of household health expenditure [16] as most Congolese are not part of any risk-sharing systems. If at all, the population of this country relies on voluntary community-based health insurance schemes [17]. Therefore, on the path towards UHC, the DRC opted for a social system based on health insurance, in which community health insurances have a predominant role [18]. This policy is an element of the healthcare reforms with the global agenda for universal access to health. In March 2016, along with other countries, the DRC agreed on a revised roadmap towards UHC in Brazzaville with a key UHC policy drawing attention to insufficient coverage in the country and to the fact that the contribution of informal sector households cannot meet the financing requirements [19].

In 2009, the Democratic Republic of Congo joined the International Health Partnership. In 2016, IHP membership was transferred to UHC 2030. On the one hand, this commitment towards UHC holds the DRC accountable to provide financial risk protection by reducing household out-of-pocket payments, which contributes to the impoverishment of many Congolese. On the other hand, the DRC government has used this commitment as a critical instrument to hold donors more accountable for their obligations.

Our study examines changes and trends over time since committing to achieve the UHC on financial risk protection and health outcomes indicators. Specifically, we examine indicators related to the second and third principles of the UHC, which aim to ensure that health services are accessible to all impoverished and vulnerable individuals; and that the country is mobilizing adequate resources for health financing. It is crucial to examine the time-related effects of different health public policies adopted by the country to understand progress towards UHC in the context of DRC’s poverty, conflicts and fragility.

Although we acknowledge the existence of other essential plans and policies like Plan Directeur de Développement Sanitaire (PNDS) 2000–2009, PNDS 2011–2015 and PNDS 2016–2020, in this research we devote much attention to the time-related effects of the DRC’s commitment towards the UHC on financial risk protection and health outcomes indicators. The three strategic plans recognize the importance for DRC to align with the recommendations of the UHC. However, DRC’s strategic plans do not clearly define how a household can easily access health services. All the strategic plans do not specify a clear policy that allows the population to access healthcare through health insurance. As a result, roughly 90% of health financing in the country originated from households out of pocket.

Hence, our study answers the following question: Is the course of financial risk protection and health outcomes indicators associated with DRC’s commitment to UHC? This study highlights the changes and trends over time since joining the UHC on financial risk protection and health outcomes indicators.

## Methods

### Study design

Universal Health Coverage is an initiative aiming to provide all people with access to needed health services. DRC joined this initiative in 2009. We collected two types of indicators to measure the progress towards the UHC. The first category of indicators measures financial risk protection, and the second measures health outcomes. The main objective of our research is to analyse whether changes have occurred in the financial protection and health outcomes indicators between 2000 and 2018. If applicable, we also analyse when these changes happened.

### Data sources

Our data originated from the World Development Indicators (WDI) of the World Bank and was complemented by the World Health Organization (WHO) database. The WDI is a compilation of comparable statistics about global development from officially international sources such as the WHO. The WHO database is a wide range of global health and well-being data sourced from their members’ states. The data collection is monitored by the World Bank (WB); the WHO data and the United Nations Inter-agency Group data are collected on an annual basis. Database closing is conducted at the end of each year with regard to the antedating year. Our study is based on data collected by the end of 2018.

Similarly, financial risk protection data are collected annually. These are aggregate data by groups computed by the WB based on the groupings for the respective fiscal year in which the data was released by WHO and WB. However, the main source of health outcomes data are vital registration systems and direct or indirect estimates based on sample surveys or censuses. This data is collected annually and compiled by the United Nations Inter-agency Group. Our research uses annual data from 2000 to 2018 to ensure balanced observation periods before and after the declaration of the DRC to adhere to UHC.

### Variables

Table 1 exhibits the variable used for our models. We retained 5 indicators to capture financial risk protection while the health outcomes component is measured by 3 indicators. Overall, we have 8 indicators that define the basis for our parametric breakpoint regression approach which is applied to estimate changes and trends over time in relation to the year when DRC joined the UHC initiative.

**Table 1:** Definition of variables

Dependent Variables				UHC Category
Acronyme	Name	Definition	Source	
OOP	Out of pocket as % of current health expenditures.	This indicator estimates how much are households in DRC spending on health directly out of pocket. It calculates the share of out-of-pocket payments over the total current health expenditures.	WHO	Financial Risk Protection
GHE	Government health expenditure as % of general government expenditure	According to the WHO, this indicator reflects the extent to which healthcare is a priority for a country.	WB	
HI	Health Insurance (CHI) as % of Current Health Expenditure	Health insurance is a combination of voluntary HI and social HI (compulsory and from the government).	WHO	
TRADOM	Transfers from domestic government revenue (allocated to health purposes), as % of current health expenditure	This indicator refers to the funds allocated from domestic government revenues for health purposes. It shows the role of central and local governments in providing revenues of health financing schemes.	WHO	
TRAFOR	Transfers distributed by the government from foreign origin, as % of current health expenditure	This indicator shows transfers originating abroad (bilateral, multilateral or other types of foreign funding) distributed through the general government. For the financing scheme receiving these funds, the provider of the fund is the government, but the fund itself is from a foreign origin.	WHO	
LRMD	Lifetime Risk of Maternal Death	The lifetime risk of maternal death is the probability that a 15-year-old girl will die from complications of pregnancy or childbirth over her lifetime; it takes into account both.	WB	Health Outcomes
PDC	Probability of dying among children ages 5–9 years (per 1000)	This indicator shows the likelihood of dying among children with ages comprised between 5 and 9 years old.		
IMMUN	Immunization, DTP3 (% of children ages 12–23 months)	*Immunization, Diphtheria, Pertussis, and Tetanus (DTP3). Child immunization measures the percentage of children ages 12–23 months who received vaccinations before 12 months or at any time before.*	WB	
Control Variables				
GDP	GDP growth (annual %)	*The Gross Domestic Product growth shows how fast the economy is growing.*	WB	
GGFC	General government final consumption expenditure (% of GDP)	*This indicator consists of expenses incurred by the government in its production of non-market final goods and services (except Gross Fixed Capital Formation) and market goods and services provided as social transfers in kind; in % of GDP.*	WB	
EDS	External debt stocks (% of GNI)	*This indicator shows in % of GNI the debt owed to non-residents repayable in currency, goods, or services.*	WB	

### Statistical methods

Data on financial risk protection and health outcomes are described using descriptive statistics of central location (mean, median), the variability (standard deviation, interquartile range), and minimum/maximum. To examine our research question, we first compared the two observation periods (2000–2009 vs. 2010–2018) for all of the outcomes using the non-parametric two-sample Wilcoxon rank-sum test [20]. This test requires at least ordinal measurements and computes the i^th^ rank of the pooled samples:

$${W}_{n_1,{n}_2}=\sum_{i=1}^{n_1+{n}_2}R\left({X}_i\right)$$

The test makes use of the U-statistic for each sample [21]:

$${U}_1={n}_1\ast {n}_2+\frac{n_1\ast \left({n}_1+1\right)}{2}-{R}_1$$

$${U}_2={n}_1\ast {n}_2+\frac{n_2\ast \left({n}_2+1\right)}{2}-{R}_2$$

Here, *R*_1_, *R*_2_ represent the sum of ranks in each sample and *n*_1_, *n*_2_ the respective number of observations. The minimum of *U*_1_, *U*_2_ is compared with critical values to accept/reject *H*_0_ : *P*(*X* < *Y*) = *P*(*X* > *Y*) vs. *H*_1_ : *P*(*X* < *Y*) ≠ *P*(*X* > *Y*). Due to ties in the data approximate *p*-values were calculated. The Wilcoxon test allows conclusions on differences in the overall distribution prior to and after the declaration of the DRC to adhere to UHC.

In addition, the parametric approach of breakpoint regression has been applied [22]. Synonyms of breakpoint regression are: change point, join point, or piecewise regression [23]. This modelling approach enables for the identification of changes in the outcome determined as trends or slopes and the location of change points [24]. Breakpoint regression has found several scientific applications: it has been applied, e.g., for the detection of changes in climate over time [25] and in time-series data [26]. One study examined annually aggregated data of suicides and found that changes of suicide methods over time coincided with governmental interventions, i.e. the results suggested strong associations of an intervention imposed on the population and the observed data on suicides [27]. To identify breaks or location(s) of changes over time, the linear predictor of a linear regression model is specified, e.g. for one breakpoint, by:

$$y={\beta}_0+{\beta}_1\ast time+{\beta}_2\ast {\left( time-\psi \right)}_{+}+\epsilon$$

Here, the breakpoint is represented by the parameter *ψ*, *β*_0_ is the model intercept, *β*_1_ the slope of time, and *β*_2_ the difference in slopes introduced by a change of an association over time. The latter is computed by (*time* − *ψ*)_+_ = (*time* − *ψ*) × *I*(*time* > *ψ*) with *I*(·), the indicator function, being one if time is greater than *ψ* the breakpoint. We used the R package *segmented* [28] in our study which estimates the optimal breakpoint in an iterative approach requiring only a vector of start parameters for *ψ*_*start*_, the length of this vector corresponds to the number of assumed breakpoints. Several linear models are then fitted until convergence to find the optimal estimate of a breakpoint $\hat{\psi}$ [28]. To define the optimal number of breakpoints the use of the bayesian information criteria $BIC=-2\mathit{\ln}\left(\mathcal{L}\right)+k\ast \mathit{\log}(n)$ [29] is recommended for breakpoint regression [28, 30]. Only estimates and respective confidence intervals will be provided for the parametric approach as the computation of *p*-values has been shown to be slightly biased [28]. For all outcomes, we compared models using none, one, two, or three breakpoints and presented adjusted R squared as well as BIC statistics. All statistical analyses were conducted using R version 4.1.1 [31].

## Results

### Descriptive statistics

Except for the Lifetime risk of maternal death (LRMD, Table 2), the data were completely available for all outcomes and indicators. For most measurements, skewed distributions were found when comparing locations of mean and median (Table 3).

**Table 2:** Description of variables

Variables	Definition	n	Mean	Median	St dev	Min	Max	Q1	Q3	IQR
OOP in %	Out of pocket	18	55.9	46.4	16.3	37.2	79.0	39.4	69.4	30.0
GHE in %	Government Health expenditure	18	3.0	2.5	0.8	2.5	4.7	2.5	3.8	1.4
HI in %	Health Insurance	18	1.3	0.0	1.8	0.0	4.8	0.0	3.0	3.0
TRADOM in %	Government transfer to health from domestic revenue	18	8.1	7.0	4.6	2.6	16.1	5.2	13.8	8.6
TRAFOR in %	Government’s transfer to heath from foreign origin	18	12.6	15.8	9.8	0.0	27.8	9.7	22.5	12.8
LRMD in %	Lifetime risk of maternal death	17	4.0	3.7	1.0	2.7	5.9	3.2	4.4	1.1
PDC in %	Probability of dying among children	18	17.2	16.4	3.4	11.9	23.1	14.3	18.6	4.3
IMMUN %	% of immune children	18	50.9	60.0	18.8	18.0	75.0	54.0	68.0	14.0
GDP %	Annual growth of GDP	18	3.4	6.1	4.5	−6.9	9.5	2.9	6.9	4.0
GGFC %	Government final consumption in % of GDP	18	6.6	6.9	1.6	2.1	9.1	5.9	8.2	2.3
EDS	External debt stock in % of GNI	18	92.5	63.1	88.7	9.6	283.9	16.7	93.3	76.6

**Table 3:** Financial risk protection and health outcomes before and after joining the UHC by DRC

Outcomes	Median: 2000–2009	Median: 2010–2018	Difference in location	Confidence interval	*p*-value
OOP	68.83	39.36	−29.41	[−32.28; − 14.38]	< 0.001***
GHE	2.49	3.84	1.35	[0.49; 1.42]	< 0.001***
HI	0.00	2.97	2.97	[1.61; 3.80]	< 0.001***
TRADOM	5.24	13.84	7.86	[5.34; 9.91]	< 0.001***
TRAFOR	10.09	22.51	13.02	[7.23; 18.86]	< 0.001***
LRMD	4.25	3.28	−1.02	[−1.50; −0.55]	< 0.001***
PDC	18.35	14.30	−4.10	[−5.40; −2.70]	< 0.001***
IMMUN	57.00	63.00	9.87	[−3.00; 27.00]	0.141

Median values for financial risk protection and health outcomes indicators before and after DRC’s commitment to UHC. Significance of differences tested using Wilcoxon rank sum test *** *p* < 0.01, ** *p* < 0.05, * *p* < 0.1

**Fig. 1:**
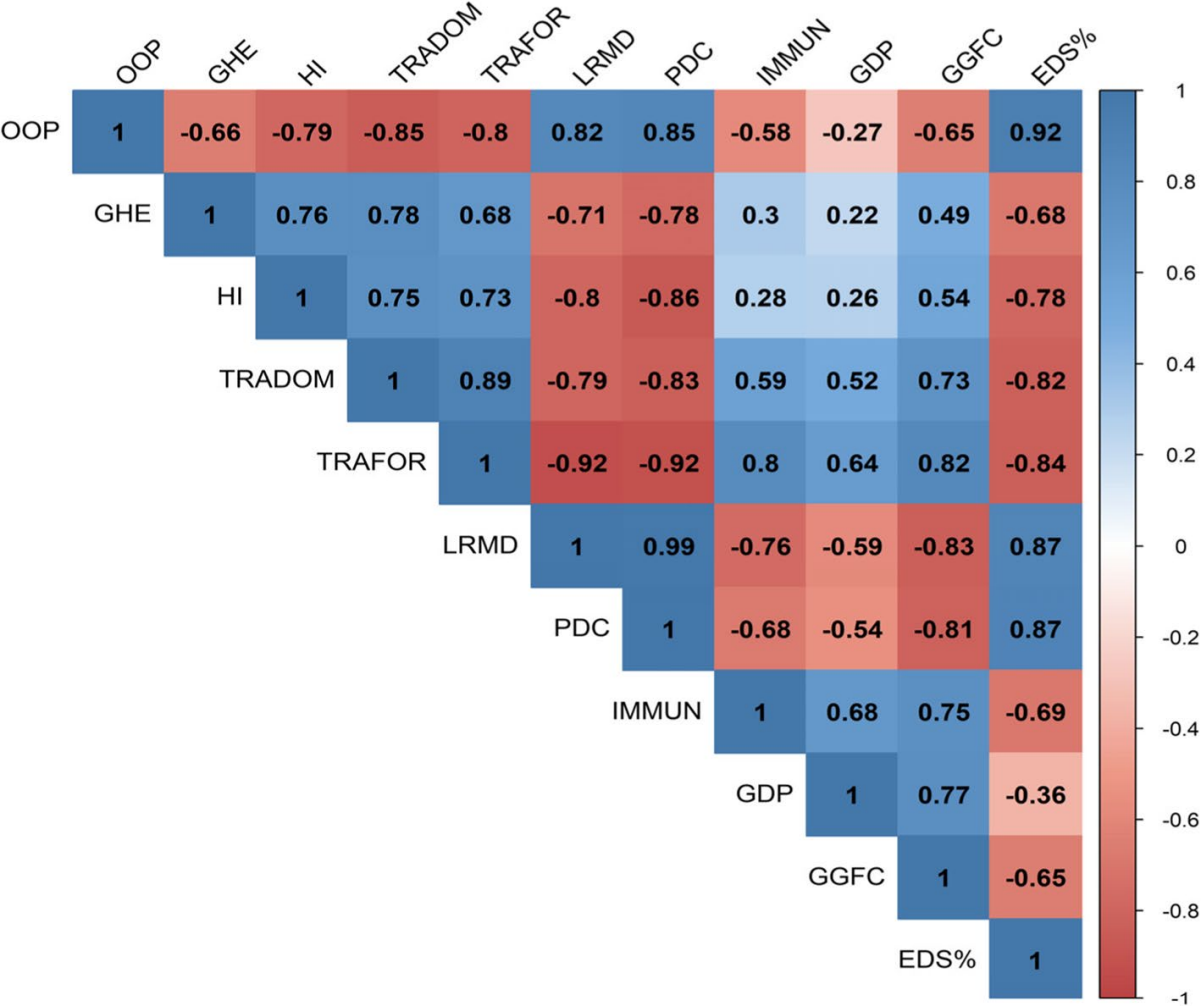
Bivariate correlations of outcomes and covariates

Figure 1 shows the degree of linear dependence between our variables. We observe mostly very strong correlations (*ρ* > 0.5) among our variables. To avoid the problem of collinearity in the parametric approach and to ensure the independence of our predictor variables, we only consider GDP as a possible covariate additionally to time.

Table 3 presents the results of the Wilcoxon rank-sum test. A decrease in Out of Pocket (OOP) from 68.83 to 39.36% has been found which presents a considerable difference also statistically significant at the 1% level. Decreases were also found for the variable LRMD (from 4.25 to 3.28%) and the outcome PDC (18.35 to 14.30%), both differences are significant at 1%. Moreover, both variables showed a strong linear association (Fig. 1) supporting a simultaneous change.

Regarding the variable GHE, Table 3 shows an increase in the proportion of funds allocated to the health sector from 2.49 to 3.84%. This result is also significant at the 1% level. Similarly, we found increases after 2009 for HI, TRADOM, as well as TRAFOR, all of these changes are significant at the 1% level. For the variable IMMUN, the positive change in the percentage of immune children was not statistically significant and has increased from 57 to 63%.

#### Results from breakpoint-regression

We use the Bayesian Information Criteria (BIC) to determine the best number of breakpoints for each outcome (Table 4). The best model for each outcome, according to the BIC, was similar to the model with the highest value of adjusted R square. Therefore, our results suggest that all outcomes are not subject to linear trends over time. A single breakpoint has been estimated for the variables GHE; HI, as well as TRAFOR. A model with two breakpoints was estimated for the variable OOP, TRADOM, and the variable PDC. Finally, only the variable LRMD and the variable IMMUN suggest a model with three breakpoints. Among all our outcomes, only the variable IMMUN yields strong results when adjusted for GDP. This shows a strong influence of GDP on the percentage of immune children. The rest of our outcomes support models without adjusted GDP. Column 2 indicates if the breakpoint regression model was adjusted for GDP (yes/no).

**Table 4:** Model selection: best number of breakpoints

Outcomes	Adjusted for GDP	adj. R2 (LM)	adj. R2 (1 BP)	adj. R2 (2 BP)	adj. R2 (3 BP)	BIC (1 BP)	BIC (2 BP)	BIC (3 BP)	Best no. of BPs
OOP	no	0.692	0.769	0.928	0.920	138.251	**119.153**	124.060	2
OOP	yes	0.704	0.859	0.932	0.926	130.462	119.538	123.554	2
GHE	no	0.658	0.806	0.658	0.756	**20.108**	27.318	30.314	1
GHE	yes	0.692	0.792	0.692	0.766	22.993	27.144	30.654	1
HI	no	0.785	0.900	0.888	0.785	**38.595**	44.011	49.673	1
HI	yes	0.824	0.894	0.824	0.824	41.492	47.703	47.703	1
TRADOM	no	0.704	0.740	0.879	0.704	94.456	**83.041**	93.403	2
TRADOM	yes	0.695	0.803	0.878	0.869	90.819	84.643	88.381	2
TRAFOR	no	0.704	0.884	0.874	0.704	**105.044**	109.806	119.297	1
TRAFOR	yes	0.752	0.878	0.864	0.883	107.594	112.611	112.118	1
LRMD	no	0.964	0.995	0.995	0.999	−49.608	−44.746	**−82.680**	3
LRMD	yes	0.971	0.997	0.999	0.999	−56.102	−80.789	− 82.265	3
PDC	no	0.999	1.000	1.000	0.999	−64.350	**−79.153**	−32.198	2
PDC	yes	0.999	1.000	1.000	1.000	− 77.672	−78.161	−72.846	2
IMMUN	no	0.317	0.815	0.791	0.317	129.292	134.732	150.577	1
IMMUN	yes	0.482	0.802	0.842	0.886	132.225	130.824	**127.156**	3

*BP* breakpoint, *adj. R2* adjusted R squared, *LM* linear model, *BIC* bayesian information criterion, **bold text** = preferred model

**Fig. 2:**
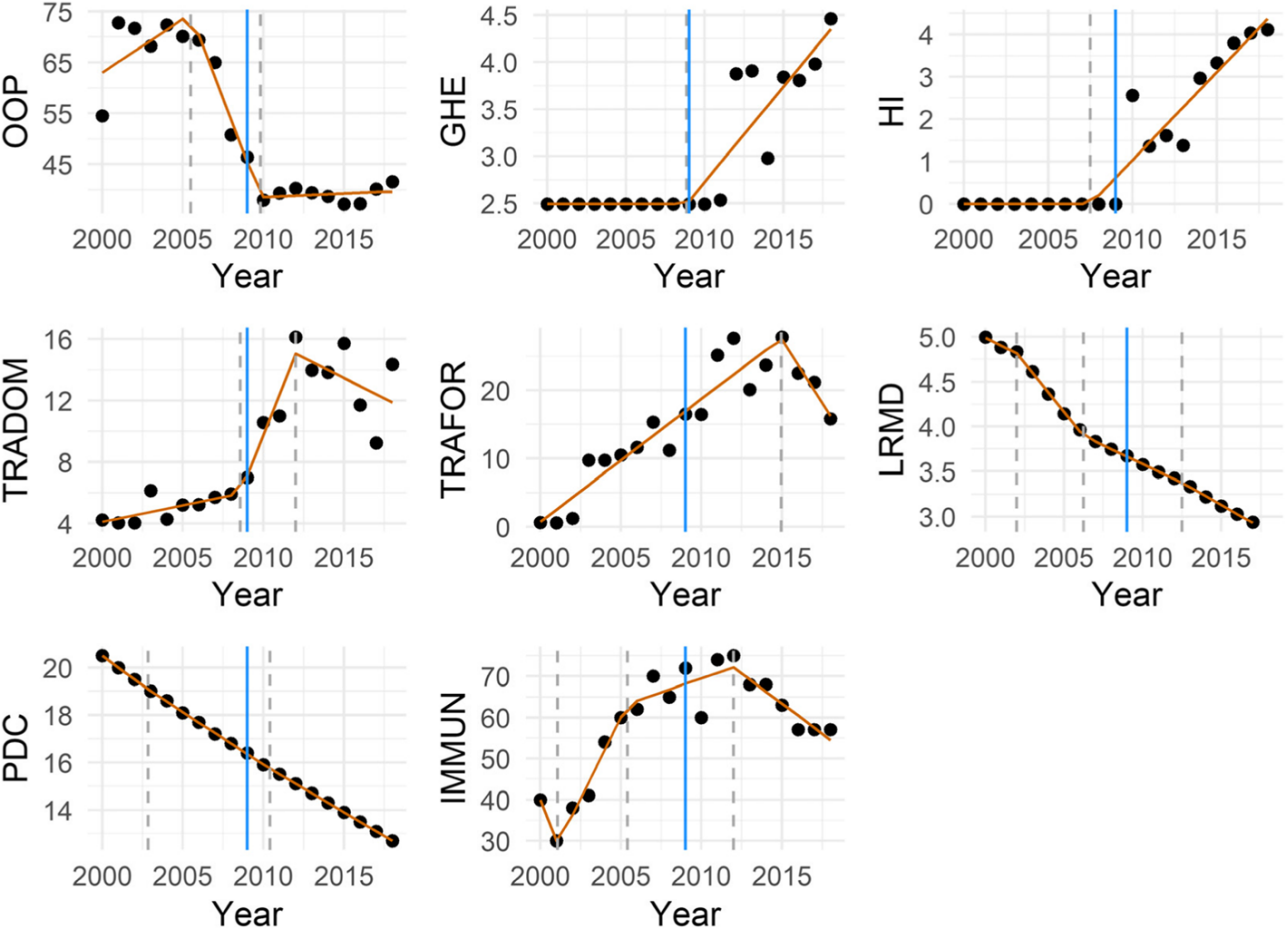
Best models: breakpoints estimation for financial risk protection and health outcomes indicators*. * This figure illustrates the course of all outcomes over time. On the y-axis the percentage of respective outcomes is found, the x-axis refers to time measured in years. Dashed vertical lines (grey) indicate the estimated breakpoints and the blue vertical line indicates the year of DRC’s commitment to UHC

After the selection of the best number of breakpoints for each outcome, Table 5 shows the results of the parameters estimated using the breakpoint regression. Our approach estimates the direction and magnitude of changes in the outcomes that were found for different time-periods separated by the estimated breakpoints. For the OOP variable, a strong decrease of 8.3% per year from 2005 to 2009 was found (Table 5, Fig. 1), i.e. before the DRC declared adherence to the UHC. During the Post-2009 period, we observe a slight increase in the OOP but this increase is not statistically significant. Hence, results from the parametric approach, affirm considerable changes for OOP (Fig. 2). However, changes have set in earlier than 2009. Prior to 2005 and after 2009, the annual data of OOP were almost stationary although on different levels.

**Table 5:** Model parameter estimation

Outcome	Segments	Segment slope	Slope estimate	Confidence interval	Range
**OOP**	**1**	**slope1**	**2.099**	**[0.08; 4.12]**	**2000–2005.5**
	**2**	**slope2**	**− 8.332**	**[− 12.11; − 4.55]**	**2005.5–2009.8**
	**3**	**slope3**	**0.141**	**[− 0.95; 1.23]**	**2009.8–2018**
**GHE**	**1**	**slope1**	**0.000**	**[−0.09; 0.09]**	**2000–2008.8**
	**2**	**slope2**	**0.203**	**[0.13; 0.28]**	**2008.8–2018**
**HI**	**1**	**slope1**	**0.000**	**[−0.17; 0.17]**	**2000–2007.5**
	**2**	**slope2**	**0.415**	**[0.31; 0.52]**	**2007.5–2018**
**TRADOM**	**1**	**slope1**	**0.215**	**[−0.21; 0.64]**	**2000–2008.6**
	**2**	**slope2**	**2.649**	**[1.19; 4.11]**	**2008.6–2012**
	**3**	**slope3**	**−0.531**	**[−1.31; 0.25]**	**2012–2018**
**TRAFOR**	**1**	**slope1**	**1.800**	**[1.43; 2.17]**	**2000–2014.9**
	**2**	**slope2**	**−3.732**	**[−6.53; −0.94]**	**2014.9–2018**
**LRMD**	**1**	**slope1**	**−0.084**	**[−0.11; − 0.06]**	**2000–2002**
	**2**	**slope2**	**− 0.219**	**[− 0.23; − 0.2]**	**2002–2006.2**
	**3**	**slope3**	**−0.083**	**[− 0.09; − 0.07]**	**2006.2–2012.5**
	**4**	**slope4**	**− 0.098**	**[− 0.11; − 0.09]**	**2012.5–2018**
**PDC**	**1**	**slope1**	**−0.500**	**[− 0.53; − 0.47]**	**2000–2002.9**
	**2**	**slope2**	**− 0.442**	**[− 0.45; − 0.43]**	**2002.9–2010.4**
	**3**	**slope3**	**−0.400**	**[− 0.41; − 0.39]**	**2010.4–2018**
**IMMUN**^**a**^	**1**	**slope1**	**−9.841**	**[− 25.77; 6.09]**	**2000–2001.1**
	**2**	**slope2**	**7.895**	**[3.19; 12.6]**	**2001.1–2005.4**
	**3**	**slope3**	**1.360**	**[−0.53; 3.25]**	**2005.4–2012**
	**4**	**slope4**	**−2.954**	**[−5.82; −0.09]**	**2012–2018**

^a^the linear model has been adjusted for time and GDP

The slope estimates indicate the direction of change within respective time-periods (segments). The range of respective segments is shown in the last column. Note: the breakpoint regression approach considers time as a continuous variable and therefore estimated breakpoints do not reflect discrete numbers.

Figure 2 reveals only one breakpoint for the variable GHE corresponding to the post adherence period. The General Health Expenditure (GHE) as a percentage of the General Government Expenditures depicts a positive slope of 0.2; showing a marginal improvement in the government’s funds allocated to healthcare. However, we also found an increase of the variable HI by 0.42% per year in 2007. Although this improvement is not statistically significant, we observe a slight increase in insurance uptake in DRC. Before the adherence to UHC, the trend was constant and only increased in the period after 2009. Similar results are shown for HI, which increases during the period post-2009 with a changing point in 2007.

For both, TRADOM and TRAFOR, Fig. 2 shows consistent increases for more than 10 years starting in 2000. We observe two breakpoints for TRADOM and only one breakpoint in 2015 for TRAFOR. The sharp and constant increase of TRADOM started in 2008 and declined from 2012 onwards. Table 5 depicts the respective slopes for both variables. We observe a sharp increase in TRADOM of annually 2.6%, which started 1 year prior to UHC’s commitment. In 2012, TRADOM has significantly decreased by 0.5%. Surprisingly, in 2014, the variable TRAFOR decreased to an average point of 3.7% per year and is statistically significant. However, this indicator increased by 1.8% in 2000.

Overall, LRMD decreased significantly over the entire observation time. Figure 2 also shows that changes have set in earlier than 2009. Although the variable LRMD has decreased over the period, this improvement was not aligned with the declaration of adherence to UHC. The same pattern has been observed for PDC. The probability of dying among children decreased by 0.4% per year from 2002. We also have found a decrease in 2010 of the same magnitude for this variable prior to UHC’s adherence.

The variable IMMUN is the only outcome adjusted for GDP with 3 breakpoints. The first change of 7.9% has set in 2011 and is statistically significant which supports an improvement in the number of immune children prior to 2009. In 2005, the variable IMMUN has improved by 1.4% but this change is not statistically significant. Finally, we have identified a decrease of 2.9% in 2012 corresponding to the period post-2009.

## Discussion

### Out of pocket and health insurance

Although the trend of the variable OOP is positive during the post-2009 period, our results suggest that this increase is not statistically significant. Before 2009 there was a strong decrease in the variable OOP from roughly 70 to 40%. After 2009, OOP appears stationary at roughly 40% with a marginal positive trend over the post-2009 period. This considerable change in OOP before 2009 may have multiple reasons, e.g., it reflects also the benefits of dozen of international initiatives addressing health care in the DRC [32]. Further, such change can also be associated with the reliance ofDRCs’people on auto-medication or non-professional treatment [33].

Over the last decade, several health-financing mechanisms have been developed in DRC to avoid catastrophic health expenditure. However, those mechanisms mainly cover formal employees such as government employees who are partially covered by the government’s budget. Some employees from the formal private sector are covered by their employers as defined by the labour laws. Lastly, few community-based health insurances have also been developed [34]. However, it is important to note the lack of initiatives that promote health insurance among the poorest who represent roughly 73% of the population as estimated by the World Bank in 2018.

Moreover, in DRC, only a minor segment of the population did not finance directly their health expenses. A report from *les comptes Nationaux de la santé* shows that, respectively, 6 and 3% of the population did not have to pay out of pocket to cover their health expenditure in 2010 and 2013. Overall, the poorest in Africa have disproportionately less access to health services and are more exposed to impoverishing expenditure than non-poor people. As a case in point, in DRC, only 0.7% of men and 1% of women from the poorest quintile report receiving health insurance services while 12% of men and 15.3% of women from the wealthiest quintile have declared to benefit from health insurance [35].

Regarding the health insurance coverage, we found a positive effect in the uptake of health insurance after the DRC adhered to the UHC. This indicator presents a positive trend during the period post-2009. However, much effort should be undertaken to ensure substantial financial protection for households. A study conducted in 59 countries by [8] shows that reducing reliance on OOP payments is essential for financial protection as a 1% increase in the proportion of total health expenditure from OOP payments is associated with an average increase in the proportion of household facing catastrophic payment of 2.2%. Similarly, the WHO found that it is only when direct payment falls to 15–20% of total health expenditures does the incidence of financial catastrophe and impoverishment fall to negligible levels [36]. In DRC, over the period under this study, the average values of OOP and HI as a percentage of health expenditures are respectively 55.9 and 1.3%.

### Government health expenditures

Our findings indicate a slight improvement in the variable GHE after the adherence of DRC to the UHC. Before the adherence to UHC, the trend was constant and only increases in the period after 2009. Our results show that, on average, the DRC’s government has spent only 3% on health with a per capita health expenditure of USD 13 [16]. This result indicates that DRC’s government did not commit to meeting its engagement to reach at least 15% of annual budgets to improve the health sector as stipulated in the Abuja Declaration [37]. Although there is no consensus to determine the funding needs for UHC, a recent study from [38] showed that targets that aim to include 5% of GDP, 15% of government expenditure and USD 86 per capita, are interesting benchmarks. Almost all the DRC’s health strategic plan report poor governance, lack of commitment and weak institutions as the main obstacles to achieving the country’s health target. Therefore, an improvement of public financial management together with a systematic approach of implementation of reforms should be undertaken for better health spending.

### Government’s transfer to health from domestic and foreign origin

Our results suggest an improvement in the government’s transfers from domestic origins while the government’s transfers from foreign sources decrease post-2009. In 2008, the funds allocated to health from domestic origins significantly increased by 2.6%. a downward trend is observed in 2012 with a statistically significant slope of 0.5%. This trend shows a lack of consistency in setting health as a priority for the country. In their studies, [39] show that many countries lag behind the Abuja target as they continue spending on debts. As a point in case, over the past decades, East and Southern Africa paid an average of USD 14 per capita annually in debt servicing which is much more than their average per capita spending on health [40]. Moreover, there is no sufficient evidence between external and internal financing for health spending. In some countries, increased external funding was not associated with falling shares of government spending on health, and in fact, the opposite has occurred [39].

In many African countries, donors are an essential funding source. However, donor funding is often unreliable and unsustainable in the long term. Our results show similar evidence as the flow of funds from foreign origins is unstainable to help DRC to reach the UHC. The WHO suggested mandatory pre-payment financing mechanisms as the core of domestic healthcare financing, including tax and other government revenue [36].

While some countries have succeeded in closing the funding gap from domestic sources, others will continue to require sustained health financing reforms and development assistance. However, government or individual contributions should be viewed as essential health funding. In this regard, a fiscal policy choice that improves tax collection efficiency and compliance is vital for health financing [38].

### Progress in health outcomes

Overall, we observe a slight improvement in the health outcomes variables. The progress in maternal death (variable LRMD), as well as in childhood mortality (variable PDC) have set in before 2009. No changes observed for these two variables are statistically significant. LRMD has decreased from roughly 4 to 3% while the PDC decreased from roughly 18 to 14%. These changes could be attributable to important investments from the private sector and NGOs in building hospitals and healthcare in remote areas, but there is no data available to substantiate that.

Although there is an important improvement in the percentage of immune children, the difference before and after 2009 is not statistically significant for the variable IMMUN. However, the change of this variable has set in before 2009 with a statistically significant slope of 7.9%. The upward trend continues in 2005 with a slope of 1.4%. However, in 2012, this variable decreases significantly with a slope of roughly 3%.

A study from [38] confirms that sub-Saharan Africa’s health challenges are numerous and wide-ranging to the extent that health outcomes are worse in fragile countries, rural areas, urban slums, and conflict zones among the poor, disabled and marginalized people. This result supports DRC’s context, which accounts for 54% of the rural population, and 73% of the poverty rate considering the threshold of USD 1.90 of consumption per day. In Africa, the maternal mortality ratio remains at a very high frequencies of around 400 per 100.000 live births, with the most significant challenge being neonatal mortality [38]. In the same study, [38] concluded that it will take more than 110 years before African newborns have the same chance of survival as newborns in high-income countries suggesting efforts above and beyond the Millennium Development Goal are necessary to reduce the maternal mortality ratio to less than 70 per 100.000 live births.

According to [41], DRC was the third-largest recipient of the Global Alliance for Vaccines and Immunization (GAVI) funds. Although we recognize that such initiatives have fostered progress in immunization performance, our results suggest that this effort should continue in order to meet the set goal for UHC. In the same spirit, [41] found that GAVI support has increased DTP3 coverage and immunization, but the government should undertake additional efforts to ensure the sustainability of routine immunization programs.

### Limitations

This study is based on publicly available data provided by the WHO Worldbank. The institution mentions high quality of the data published, however, also acknowledges challenges in data acquisition in fragile states [42]. The quality of data used in this study rests on administrative capacities of the DRC which might still be to restricted. This may also affect the validity of provided data due to limited monitoring of the data collection process. It is therefore possible, that some trends and changes found in this study might be biased upwards or downwards. Nevertheless, the key message of this study will be unaffected as the DRC has to increase its efforts to comply with UHC.

## Conclusion

DRC’s UHC journey started in 2009. This paper has examined the changes in financial risk protection and health outcomes indicators during a 9-years interval (2010–2018) after DRC has committed to UHC. We implemented the parametric approach of breakpoint regression to detect whether the UHC journey has brought changes and when exactly the changes have occurred. Although OOP has improved, the results from the breakpoint regression support an adverse effect of DRC’s commitment toward UHC on household direct spending on health. Health insurance coverage is still deficient in DRC; we observe positive effects on health insurance coverage with progress over time. DRC’s government should invest a lot in improving the conditions of the health insurance market by defining new health insurance programs while setting norms and regulations for the overall system. The effect on health insurance and OOP reliance is minor due to the adoption of initiatives that focus on and favour wealthier people than the poorest, which roughly represent 73% of the population.

Our results suggest an improvement in the government’s transfers from domestic origins while the government’s transfers from foreign sources decrease. In 2008, the funds allocated to health from domestic origins increased up to 2012 when a negative breakpoint is identified. This trend shows a lack of consistency in setting health as a priority for the country.

The result of this study supports the target defined by the Abuja declaration as we found that DRC’s government does not allocate sufficient funds towards achieving UHC. While the increase in the government’s transfers to health from domestic origins is not sustainable, the effect on the transfers from foreign sources was not identified, suggesting that either the government did not properly allocate donors’ funds or donors did not meet their commitment.

Overall, the risk of maternal death has decreased significantly. Similar results appear for the probability of dying among children. However, the changes observed post-2009 are not statistically significant. These changes might be attributable to important investments from the private sector and NGOs in building hospitals and health centres in remote areas.

There is no conclusive effect on the indicator related to children who received vaccinations. In DRC, the vaccination rate of children mostly originates from donor initiatives such as GAVI, which has supported access to the vaccine among children in the country. These donor initiatives are important but mostly unsustainable and unpredictable. Moreover, DRC’s government should undertake additional efforts and provide infrastructure like electricity, roads, and refrigerators in short supply to deliver vaccines to remote health centres.

Therefore, our work suggests that although DRC’s UHC journey has slightly contributed to improving the financial risk protection and health outcomes indicators, much effort should be undertaken. In general, we make an essential empirical contribution that is relevant to the development and public policy towards the achievement of UHC. Using the case of DRC, we illustrate that many developing countries adhere to international initiatives but do not align their national policies to meet and monitor progress toward the set global agenda.

Our results reveal that the prevention of catastrophic health expenditure is still not a priority for the country and mostly for the majority of the poorest even after the DRC’s UHC journey has started. Hence, as we are approaching 2030, DRC’s government should pour into a well-structured and realistic operational plan the target goals to reduce the household’s financial burden of health expenses on households with a clear focus on the informal sector and poor households. The operational plan should focus on access to health insurance policies, which the country lacks. Achieving Universal Health Coverage in the Democratic Republic of Congo will require a set of differentiated policies for social groups. It is obvious that the poorest will not be able to pay out-of-pocket fees or an insurance premium. Thus, it might require mobilizing additional revenues to extend health coverage to the poorest. There is growing awareness that the “poorest of the poor” definitely require national (and international) solidarity. However, in DRC the majority of people are near-poor, i.e., they do not belong to the “poorest of the poor” but are constantly at risk of falling back into extreme poverty once an adverse event strikes them. For instance, the majority of the rural population of DRC works in the informal sector or in subsistence farming. Under “normal conditions” they can survive, but even a mild disease of the bread-earner of a major disease of a family member might bring them back beyond the poverty line. These people might be able to pay a token for social protection, but they will definitely also require a subsidy of their social protection contribution. To generate additional revenue to accelerate the agenda of UHC, the country should invest more in an effective tax collection system. With the additional revenue, the government could define an incentive programme to subsidize health insurance and healthcare for the majority of the poorest and the near-poor in the informal sector and rural areas.

For coverage expansion of the wealthy quintile of the population and the minority of the middle class, mandatory health insurance might be an option in the long term and should help to generate additional funds to support the government incentive programme.

The use of technology to support health policies should also be prioritized towards achieving UHC as the country faces logistical and infrastructure issues to monitor UHC’s progress in a vast country of 0.23 times as big as Europe. Setting technology at the centre of every health policy will enable the country to collect important data, which mostly are not available at the national level. Therefore, the country can rely on available data to promote information sharing and build accurate policies. Finally, DRC’s government should implement appropriate health insurance policies through well-organised social health insurance (voluntary community-based health) as many Congolese are involved in the informal sector to reduce OOP spending. The government can also subsidise hospital services for the poor. Implementing a robust monitoring and evaluation strategy will also ensure that the household’s health outcomes are improved as the country strives to implement appropriate and context-based health insurance regulations.

## Data Availability

The datasets analysed during the current research are available from the corresponding author upon request.

## Abbreviations

BIC: Bayesian Information Criteria
DRC: Democratic Republic of Congo
GAVI: Global Alliance for Vaccines and Immunizations
GDP: Gross Domestic Product
HI: Health Insurance
IHP: International Health Partnership
NGO: Non-Governmental Organisation
OOP: Out of Pocket
PNDS: Plan Directeur de Développement Sanitaire
UHC: Universal Health Coverage
USD: United States Dollar
WB: World Bank
WDI: World Development Indicators
WHO: World Health Organization
